# pREBOA Abroad: Leveraging Partial Aortic Occlusion for Stabilization and Transport in Ukraine

**DOI:** 10.7759/cureus.84466

**Published:** 2025-05-20

**Authors:** Eric Akrish, Courtney H Meyer, Volodya Spokiy, Erin C Caddell, Jonathan Nguyen

**Affiliations:** 1 Emergency Department, Tampa General Hospital, Tampa, USA; 2 Surgery Department, Emory University School of Medicine, Atlanta, USA; 3 Surgery Department, Ukrainian Armed Forces, Kyiv, UKR; 4 Surgery Department, Grady Memorial Hospital, Atlanta, USA; 5 Surgery Department, Morehouse School of Medicine, Atlanta, USA

**Keywords:** austere, partial aortic occlusion, partial reboa, preboa, prolonged field care

## Abstract

The war in Ukraine highlights the challenges in providing combat casualty care when both nations are near-peer adversaries. Providing timely definitive hemorrhage control within one hour of injury, as the US military and its allies have grown accustomed, is unrealistic. Stabilization points (SP) in Ukraine are forced to provide prolonged field care to exsanguinating patients with little resources while waiting extended periods of time for patient transport. pREBOA-PRO™ (Prytime Medical, Boerne, TX)has been suggested as a bridge to this problem as it can provide temporary hemorrhage control, stabilize the patient, and limit distal ischemic consequences when used for partial aortic occlusion. We discuss a patient who suffered a traumatic arm amputation and intra-abdominal injuries managed with pREBOA-PRO™. This partial aortic occlusion provided an increase in central perfusion pressure, temporary hemorrhage control, and time for patient transport to definitive hemorrhage control. This serves as proof of concept that pREBOA-PRO™ and partial aortic occlusion can stabilize a patient, provide longer safe occlusion times, and aid in transport in austere environments while mitigating distal ischemic consequences.

## Introduction

In an effort to improve time to definitive hemorrhage control and mortality, the US military adopted the principle of the "golden hour" to ensure timely definitive hemorrhage control for wounded individuals [1]. With the aid of air superiority, combat casualties would arrive at a facility capable of damage control surgery within 60 minutes from the point of injury (POI). This proved effective at reducing mortality over the past 20 years in conflicts such as Afghanistan and Iraq and has governed how resources and people were deployed in war zones [2].

However, there is growing concern that future wars and conflicts will be between near-peer adversaries with similar technology, weaponry, and capabilities, inhibiting the ability to provide prompt surgical hemorrhage control and stabilize combat casualties [3]. This is highlighted by the ongoing war between Ukraine and Russia in which large-scale combat operations, drone attacks, artillery, and matched weaponry prohibit the use of air or ground assets to quickly and safely transport injured personnel to facilities with damage control surgical capabilities [3]. Patients are instead transported to stabilization points (SP) under hazardous conditions and almost exclusively by ground. These SP have limited resources (fluids, medications, and blood) and are exceedingly close to the front line, making them vulnerable to rockets, artillery, and drone attacks. While these SP are capable of basic procedures (emergent airways, tube thoracostomy, wound packing, and field amputations), they lack the ability to achieve hemorrhage control of torso injuries. Despite these constraints, SP are tasked with obtaining temporary hemorrhage control, maintaining stability, and keeping someone alive for hours at a time with minimal resources until they can reach a damage control surgical facility.

Resuscitative endovascular balloon occlusion of the aorta (REBOA) is a procedure that utilizes a catheter to completely occlude the aorta, providing temporary hemorrhage control and stability in exchange for distal ischemia [4]. It has been utilized in the prehospital and combat environment with some success; however, safe occlusion times of <30 minutes limit wide adoption [5,6]. A new REBOA catheter, the pREBOA-PRO™ (Prytime Medical, Boerne, TX), is capable of both complete and titratable partial aortic occlusion, as the clinical situation demands [7]. This is accomplished utilizing a new semi-compliant balloon designed with small grooves along the long axis. When fully inflated, the balloon is a seamless smooth oval shape that provides complete occlusion. When the balloon is deflated, the grooves are revealed, allowing small regulated amounts of flow across the oval balloon. Though the duration of partial aortic occlusion remains undefined, Gomez et al. [7] demonstrated median zone 1 partial aortic occlusion times of 40 minutes (IQR: 25-74), and Campbell et al. [8] illustrate the ability to extend partial aortic occlusion in a gunshot wound victim to 123 minutes without distal ischemic injury [9]. In the civilian world, pREBOA-PRO™ and partial aortic occlusion have been successfully used to stabilize patients for extended periods of time with decreased blood utilization and risk of ischemic sequela [10-12].

Recognizing the unique challenges of prolonged field care and extended transport times seen in the Ukrainian war, pREBOA-PRO™ may be a plausible bridge to surgical hemorrhage control. Here, we describe one of the first uses of pREBOA-PRO™ to stabilize and transport a patient from an SP to definitive surgical intervention in Ukraine.

## Case presentation

**Figure 1:**
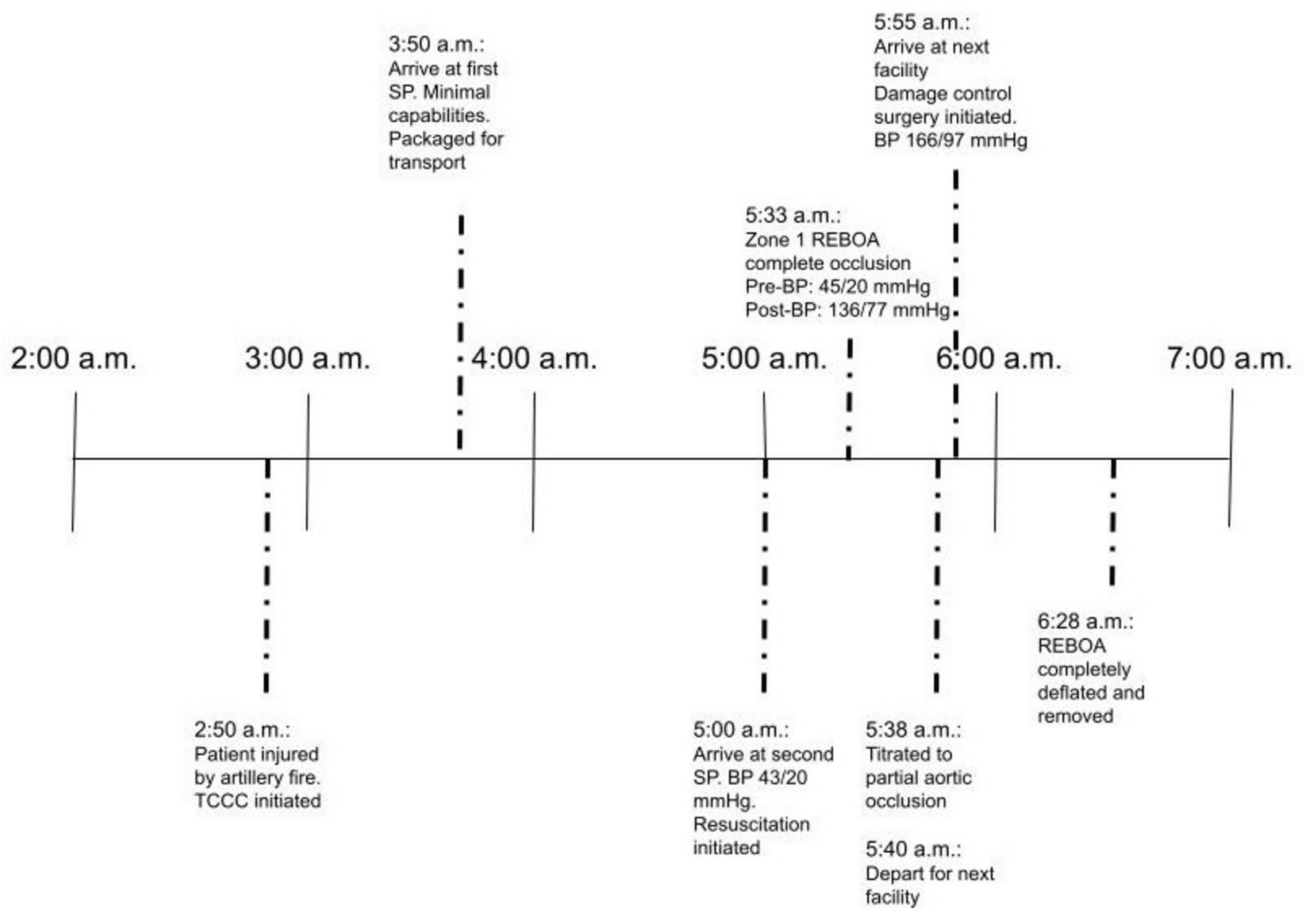
Timeline depicting events during the patient's resuscitation TCCC, tactical combat casualty care; SP, stabilization point; BP, blood pressure; REBOA, resuscitative endovascular balloon occlusion of the aorta

A 38-year-old man was struck by artillery fire at 2:50 a.m. (Figure 1). At the POI, medics provided basic wound care, and the patient was transported via ground to the initial SP at 3:50 a.m. Because of the limited resources at this SP, a bleeding abdominal wall wound was dressed, a tourniquet was assessed for hemorrhage control on an amputated arm, and the team waited for medical transport.

Because of the operational environment, he was unable to be transported to a surgical facility, and the patient was instead transported by ground to another SP, arriving at 5:00 a.m. in peri-arrest with a blood pressure (BP) of 43/20 mmHg. This SP was staffed with an emergency physician who had REBOA and partial aortic occlusion training. This SP had no damage control surgical capabilities and only four units of blood. The patient was intubated, central access was obtained, blood was initiated, and extended focused assessment with sonography in trauma (eFAST) was performed. Simultaneously, a call for transport was made. On physical examination, he had a traumatic amputation of the left arm and a disruption of the abdominal wall resulting in the evisceration of the small intestines. Hemoperitoneum was actively decompressing from the abdominal wall disruption. A common femoral arterial line was placed with an 18-gauge needle and ultrasound, confirming a core aortic pressure of 55/30 mmHg despite two units of blood. In the setting of persistent hemorrhagic shock despite blood transfusions and limited blood availability, the arterial line was upsized to a 7F sheath.

**Figure 2:**
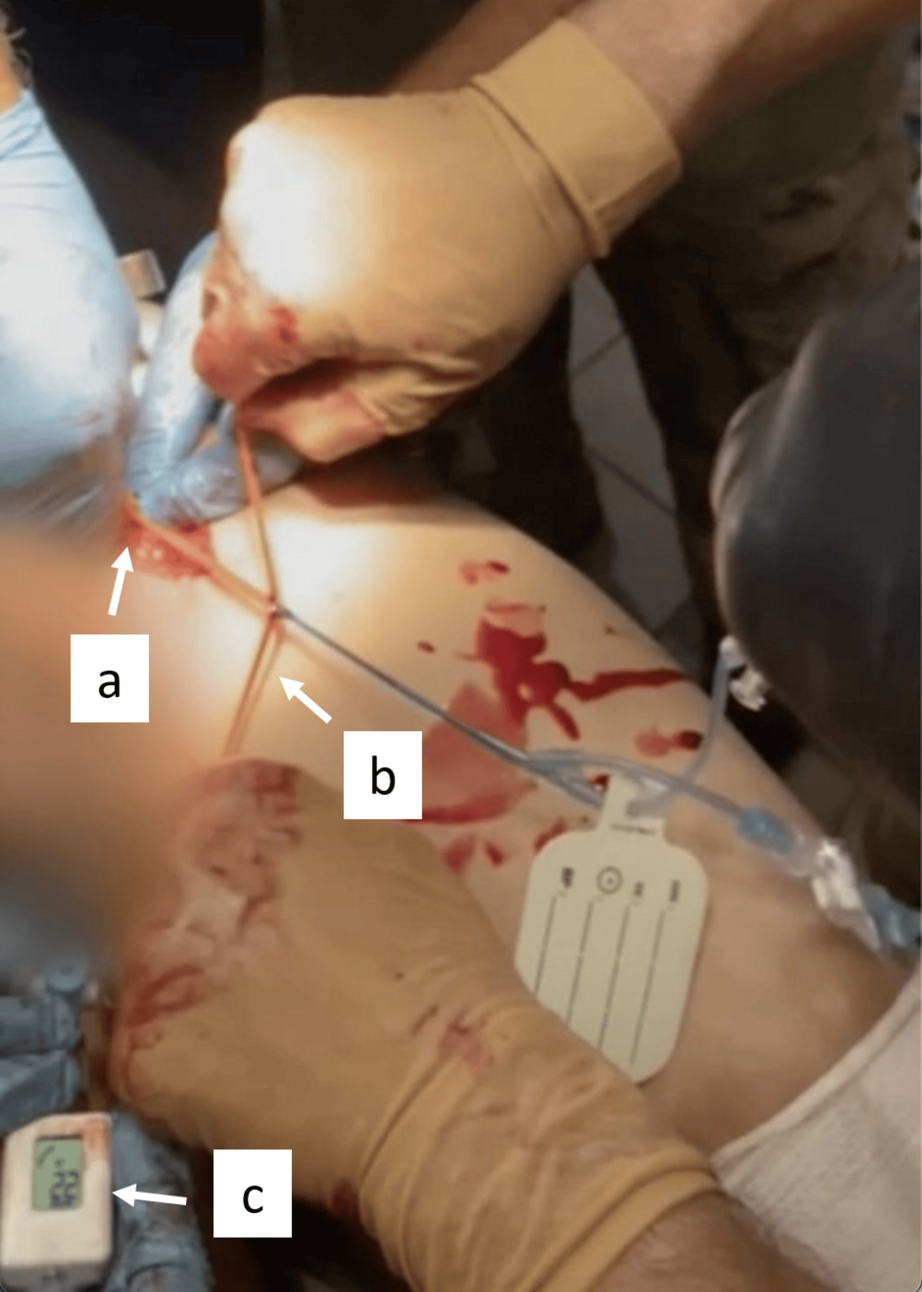
Temporary hemorrhage control achieved through partial aortic occlusion with pREBOA-PRO™ The pREBOA-PRO™ catheter is being placed into the common femoral access sheath (a), and the orange peel-away sheath, which helps advance the catheter through the 7F sheath, is being removed (b). The Compass device, an inline portable pressure monitor, has been attached to the sidearm of the 7F sheath (part of the sheath has been obscured to protect the patient's privacy). The Compass device reads a distal pressure of 22 mmHg, signifying that partial aortic occlusion has been achieved

The pREBOA-PRO™ was placed through the 7F sheath (Figure 2a) and inflated to complete occlusion in zone 1 (supraceliac) using the sternal notch as an external landmark at 5:33 a.m. Complete aortic occlusion was verified by confirming no flow through the 7F sheath's side port. The BP improved immediately from 45/20 mmHg to 136/77 mmHg (post inflation). The tourniquet was removed after ligating the brachial vessels, and two more units of blood were transfused. At 5:38 a.m., pREBOA-PRO™ was titrated down to partial occlusion without massive increased intra-abdominal bleeding or instability. Partial aortic occlusion was achieved with the aid of the Compass Universal Hg device (Centurion Medical Products, Williamston, MI), an inline portable pressure monitor, and was titrated to a distal pressure of 25 mmHg (Figure 2c: the image shows the Compass device reporting a pressure of 22 mmHg because the pREBOA-PRO™ catheter was actively being titrated at the time of image capture). The eviscerated bowel was covered with moist gauze, and the abdominal wall defect was packed. He was transported to the next echelon of care at 5:40 a.m. In total, he received two units of packed red blood cells, two units of plasma, 500 cc of normal saline, 100 cc of Gelaspan, and 2 g of tranexamic acid (TXA). En route to the hospital by ground, his vitals remained stable with a BP of 166/97 mmHg.

He arrived at a surgical hemorrhage control facility at 5:55 a.m. and was brought immediately to the operating room. On laparotomy, there was a high-grade splenic injury and injuries to the small bowel and left colon. A splenectomy, small bowel resection, and left hemicolectomy were performed, leaving him in discontinuity. There were no overt signs of ischemic injuries to the abdominal viscera. The pREBOA-PRO™ was slowly deflated during the case and completely deflated at 6:28 a.m. without complication. The total time of REBOA was 55 minutes (five minutes of complete occlusion, 50 minutes of partial occlusion, and 15 minutes of transport time). The patient was evacuated to the region's hospital for abdominal and extremity definitive surgery. As of hospital day 6, the patient was recovering in the ICU.

## Discussion

The current war in Ukraine underscores the possible new combat medical landscape in the next conflict. Medical providers may be working in a conventional wartime scenario between near-peer forces, and the golden hour of care to a damage control surgical facility may not be feasible. The use of partial aortic occlusion and pREBOA-PRO™ in a resource-limited austere environment afforded medical personnel time to stabilize the patient until transport was available without ischemic consequences.

This case depicts one of the first uses of pREBOA-PRO™ for deliberate and titratable partial aortic occlusion in a combat situation. This patient had several sources of hemorrhage, including intra-abdominal bleeding and an amputated extremity, and arrived at an SP with only four units of blood. This patient arrived in peri-arrest with a systolic blood pressure of 45 mmHg and was unresponsive to two units of blood. Without pREBOA-PRO™ as an adjunct at this SP, the patient had little chance for survival. The alternative would have included a resuscitative thoracotomy, which allows for only 30 minutes of aortic occlusion and carries an exceedingly high mortality rate. Though the actual transport time was only 15 minutes, the partial aortic occlusion gained temporary hemorrhage control, aided in stabilizing a patient in peri-arrest when blood was scarce, allowed time for the team to organize transport and safely transport the patient, and provided hemodynamic stability at the surgical facility as they began their operation and resuscitation. The success of this endeavor illustrates the potential role of pREBOA-PRO™ and partial aortic occlusion as a resuscitative adjunct in combat casualty care and other resource-limited settings by serving as a bridge to surgical hemorrhage control.

While this initial success is promising, it is only one case that demonstrates its utility. Furthermore, our case study is limited by the nature of this wartime environment, which precludes our ability to provide additional vital signs, interventions, track time variables, total resuscitation requirements, and ultimate outcomes of the patient. Additionally, while there were no obvious signs of ischemic injuries to the distal viscera, there is no quantifiable way of demonstrating this. Finally, the use of this device in the austere environment does not conclude the resuscitation and stabilization phase of a patient's care. It aids the treatment team by providing time to mobilize the resources needed for definitive hemorrhage control. There is a need for larger studies to determine the optimal efficacy and limitations of pREBOA-PRO™ and partial aortic occlusion in this setting. Equally important is a best-use guideline tailored to partial aortic occlusion and pREBOA-PRO™ in austere environments.

## Conclusions

This case study serves as proof of concept that partial aortic occlusion can be performed in an austere environment to stabilize an exsanguinating patient and provide temporary hemorrhage control for transport to a higher echelon of care. The zone 1 partial aortic occlusion time breaks conventional REBOA limits of 30 minutes, which is critical when the golden hour of combat medical transport does not exist. Further research and guidelines are required to determine the optimal patient population and the use of this procedure in an austere environment.
